# Novel Perspective on Molecular and Cellular Adaptations of the Mammary Gland-Regulating Milk Constituents and Immunity of Heat-Stressed Dairy Cows

**DOI:** 10.1021/acs.jafc.4c03879

**Published:** 2024-09-03

**Authors:** Franziska Koch, Dirk Albrecht, Elke Albrecht, Christiane Hansen, Björn Kuhla

**Affiliations:** † Research Institute for Farm Animal Biology (FBN), Dummerstorf 18196, Germany; ‡ Department for Microbial Physiology and Molecular Biology, University of Greifswald, Greifswald 17489, Germany; § Mecklenburg-Vorpommern Research Centre for Agriculture and Fisheries, Institute of Livestock Farming, Dummerstorf 18196, Germany

**Keywords:** hyperthermia, dairy cows, udder, proteomics, inflammation, adaptation

## Abstract

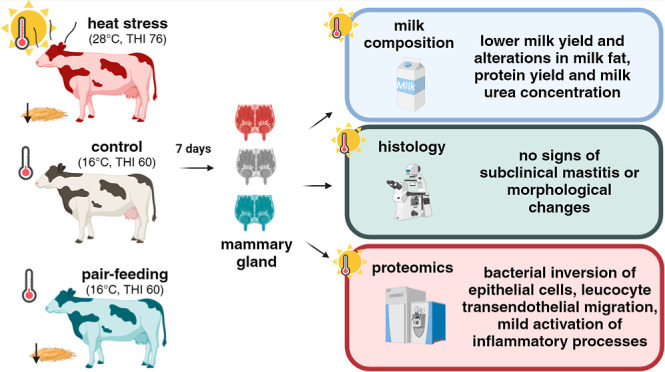

Climate change with increasing ambient temperatures negatively influences the biology of dairy cows and their milk production in the mammary gland (MG). This study aimed to elucidate the MG proteome, differences in milk composition, and ruminal short-chain fatty acid concentrations of dairy cows experiencing 7 days of heat stress [HS, 28 °C, temperature humidity index (THI) = 76], pair-feeding (PF), or ad libitum feeding (CON) at thermoneutrality (16 °C, THI = 60). Ruminal acetate, acetate/propionate ratio, and milk urea concentrations were greater, whereas milk protein and lactose were lower in HS than in control cows. Proteome analysis revealed an induced bacterial invasion of epithelial cells, leukocyte transendothelial migration, reduction of the pyruvate and carbon metabolism, and platelet activation in the MG of HS compared to CON or PF cows. These results highlight adaptive metabolic and immune responses to mitigate the negative effects of ambient heat in the MG.

## Introduction

Climate change is one of the greatest challenges facing humanity. The prediction of more frequent weather extremes and heat waves poses a thermal threat to farm animals.^1^ In particular, dairy cows are not thermotolerant and susceptible to heat stress (HS) caused by rising ambient temperatures.^2^ The switch from thermoneutrality to HS is associated with multiple effects on thermoregulation for heat dissipation, e.g., increased rectal temperature, sweating, respiration frequency, and heart rate,^3,4^ all of which compromise animal health and welfare. In order to maintain homeothermy and to minimize endogenous and fermentative heat production, feed consumption and milk yield decrease with prolonged HS.^4,5^ Milk synthesis depends on the availability of nutrients in the arterial blood, among others, glucose, acetate, long-chain fatty acids, and amino acids. HS-induced feed intake depression leads to a nutrient shortage, directly affecting the synthesis of milk constituents of the mammary gland (MG).^6−8^ However, the reduction in feed intake only accounts for ∼70% of the observed decrease in milk production, as shown by the comparison of milk yields of pair-fed (PF) cows kept at thermoneutral conditions and heat-stressed (HS) cows.^3,5^

The main precursors for the de novo synthesis of milk fat are short-chain fatty acids (SCFA), mainly acetate, for even-numbered milk fatty acids. Besides, the MG may extract long-chain fatty acids from the circulation either originating from the diet or from adipose tissue depots to be used for milk triglyceride synthesis.^9^ Milk lactose production depends on the availability of glucose; the latter is synthesized from propionate in the liver. The SCFA are formed during fermentative processes in the rumen, and their amounts and portions depend primarily on feed intake and diet composition.^10^ Different studies demonstrated that the rumen metabolism, SCFA concentrations, and absorptions are modulated during thermal challenge.^10,11^ While HS heifers showed diminished butyrate absorption when compared with PF heifers kept at thermoneutral conditions,^11^ HS cows had greater ruminal acetate, propionate, and butyrate concentrations prior to feeding than PF counterparts.^10^ However, the link between the ruminal SCFA concentration and milk composition during HS has not been comprehensively evaluated. Furthermore, it is well known that milk protein synthesis depends on the available amino acids and small peptides absorbed before from the small intestine.^12^ Milk proteins such as caseins, α-lactalbumin, and β-lactoglobulin are synthesized in the lactocytes.^13^ Of note, the effect of ambient heat on milk composition, e.g., milk protein, milk fat, and lactose, is not consistent between studies and depends on the severity and length of the heat events, but also the lactation number, milk performance, and parity of the cows are further reasons for the inconsistent findings.^14^

Besides the shortage of nutrient supply for milk production, HS seems to have direct adverse effects on MG health, including changes in somatic cell count, microstructure, and cellular processes and increased risk of mammary infections. Several studies have found a positive correlation between HS caused by a higher temperature humidity index (THI) during the summer months and somatic cell count (SCC) in individual and bulk tank samples (reviewed by Rakib et al., 2020^15^). Furthermore, conditions exceeding a THI of 60 also increase the incidences of mastitis-causing pathogen infections, e.g., by yeast and *Streptococcus uberis*, due to better growing conditions for these pathogens at higher temperatures.^16^ Thermal stress exacerbates the occurrence of mastitis and compromises immunity, thereby increasing the risk of mammary infections during summer months.^17^ Furthermore, previous studies showed that incubation of bovine mammary epithelial cells (MEC) at 40.5 to 42 °C induced a HS response and cellular repair mechanisms, while molecular pathways related to cell cycle, cell differentiation, and cell structure maintenance were inhibited.^18,19^ There is further evidence that HS alters the MG development between late lactation and involution.^20^ Transcriptomic analysis revealed down-regulated genes involved in mammary parenchymal development with a concomitantly induced thermal stress response at the cellular level in HS compared to cooled cows during summer.^20^ A preliminary proteome study including 4 cows identified alterations in the pyruvate, glyoxylate, and dicarboxylate metabolism of HS cows exposed to 32–36 °C (THI = 82–87) for 9 days compared with pair-fed (PF) cows kept at 20 °C (THI = 65).^21^ However, these data do not yet explain changes in milk composition during HS and provide no information on the cellular and immunological adaptation of the MG to thermal threat of dairy cows. Thus, we hypothesized that HS would negatively alter ruminal SCFA concentrations, milk composition, and concomitantly milk fat, protein, and lactose synthesis pathways in the MG. We further expected that HS disrupts MG metabolism and potentially activates a low-grade inflammatory response. Therefore, the objective of this study was to elucidate ruminal SCFA concentration, milk composition, and the MG proteome of thermally stressed primiparous dairy cows and compare them with control (ad libitum feeding) and pair-fed dairy cows kept at thermoneutrality for 7 days.

## Materials and Methods

### Animals and Treatments

The ethics committee of the State Government in Mecklenburg-West Pomerania, Germany, approved all treatments and procedures (LALLF no. M-V/TSD/7221.3-1.1-60/19). All experiments and methods were carried out in accordance with relevant guidelines and regulations and in compliance with the ARRIVE guidelines.^22^ Thirty primiparous, nonpregnant German Holstein cows [mean ± SD: 169 ± 48 days in milk (DIM)] were selected from the herd of the research institute and genotyped based on HSP70.1 5̀UTR SNPs.^4^ As described earlier, cows were evenly assigned to three different groups: heat-stressed (HS, *n* = 10), control (CON, *n* = 10), and pair-fed(PF, *n* = 10).^4^ In brief, all cows were moved to climate chambers, received feed for ad libitum intake, and allowed to acclimate for 6 days to thermoneutrality (TN) at permanent 16 °C and relative humidity (RH) of 69%, resulting in a temperature–humidity index (THI) of 60.^4,23^ In the climate chambers, the day–night rhythm was given by a light cycle ranging from 0600 to 1900 h. During the subsequent experimental phase, the CON group was further housed at 16 °C and 69 ± 2% RH (THI = 60) with ad libitum feeding for 7 days. Subsequently, cows of the HS group were exposed to 28 °C with 51 ± 2% RH (THI = 76 ± 0.2) for 7 days. The HS cows had ad libitum access to feed and water, both tempered to 28 °C. The PF cows were fed the amount of feed per kg body weight the HS cows ingested but were exposed to 16 °C and 69 ± 2% RH (THI = 60 ± 0.2) for 7 days. PF served as a control to eliminate the cofounding effect of reduced energy and nutrient intake of the HS relative to the CON group. Details of the total mixed ratio, dry matter intake, milk yield, rectal temperature, and respiration rate are reported by Koch et al. (2023).^4^ After 7 days of challenge, the body weight was determined, and cows were transported to the institutional slaughterhouse, stunned by a captive bolt, and killed by exsanguination. Within 10 min after death, the MG weight was measured, and MG parenchyma samples were gained from the left quarter, snap-frozen in liquid nitrogen, and stored at −80 °C until further analysis.

### Surface Temperature

**Figure 1:**
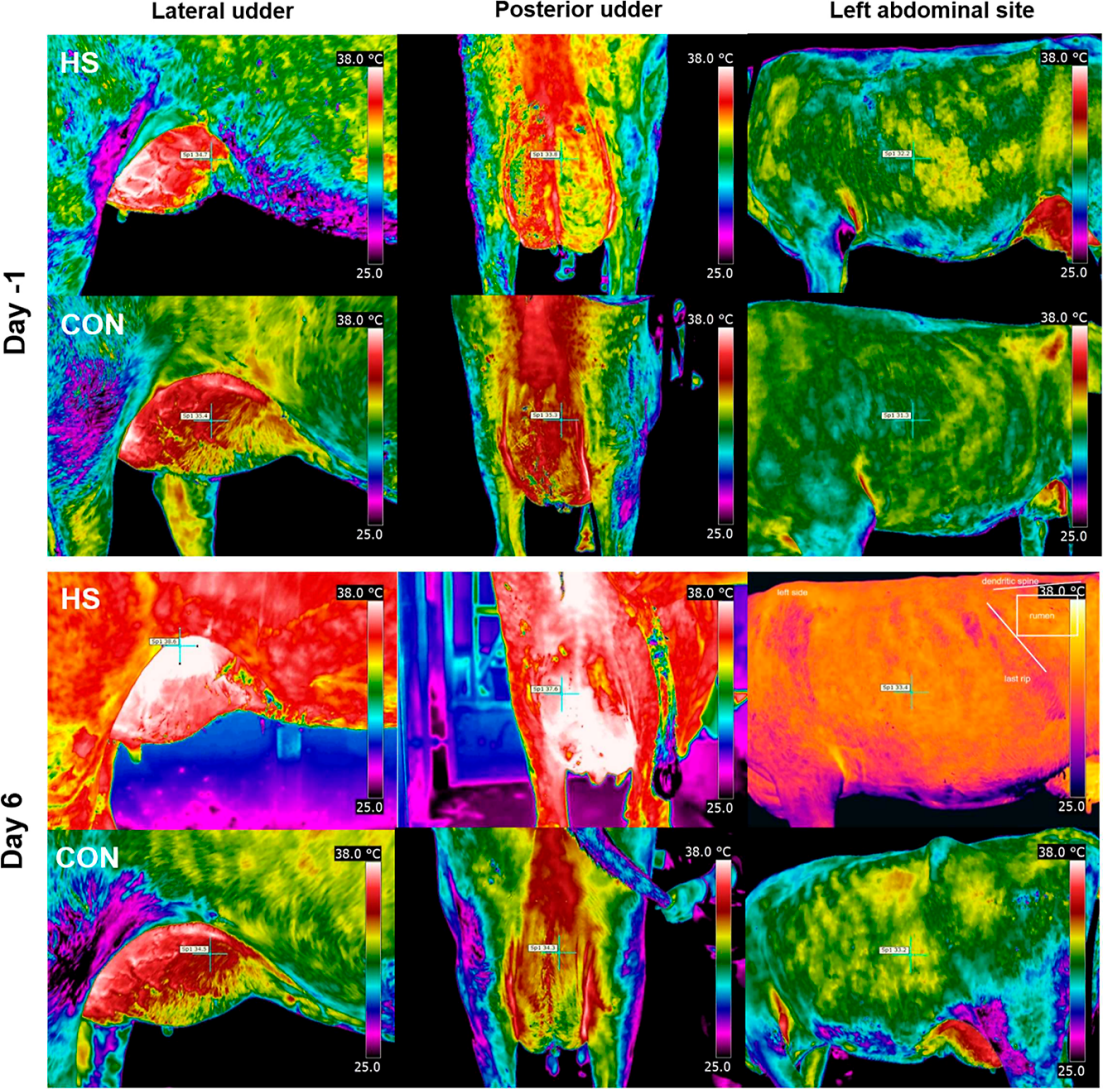
Representative images of infrared thermography on lateral and posterior MG and left abdominal site of heat-stressed (HS) or control (CON) cows on day 1 and day 6 of the 28 °C (HS) or 16 °C (thermoneutrality) exposure.

During the adaptation and experimental phases, ambient temperatures of the climate rooms were recorded every 10 min by electronic data loggers (testo 174H, Testo AG, Lenzkirch, Germany) in close proximity to the cows. One day before and again on day 6 of the experimental phase, the body surface temperature of the lateral and posterior MG side and the left abdominal site was assessed using a thermal imaging camera (T620BX, FLIR Systems, Wilsonville, OR, USA). The camera was placed parallel to the height of the area to be analyzed at a 1.5 m distance to the surface of the cow. At the infrared images of the lateral abdominal site, the area between the last rib and the dendritic spine, confining the left cranial and caudal site of the rumen, was included in the evaluation (Figure 1). The mean, minimum (min), and maximum (max) surface temperatures, as well as the difference between ambient (taken from electronic data loggers) and surface temperatures, were calculated. Due to technical issues, temperatures from only nine cows per group could be obtained.

### MG Histology

Sections of frozen MG were cut 12 μm thick with a cryostat microtome (CM3050 S, Leica, Bensheim, Germany) and stained with hematoxylin and eosin (H/E, hematoxylin: Dako, Glostrup, Denmark; eosin: Chroma Gesellschaft, Münster, Germany) following standard protocols. Images were taken using an Olympus BX43 microscope equipped with a DP23 color camera (OSIS, Münster, Germany) and CellSens imaging software (Evident, Hamburg, Germany).

### Milk Composition and Energy-Corrected Milk

Milk samples from the afternoon and next morning milking were pooled on day 2/1 before the experimental phase (day −1) and on days 1/2, 3/4, and 5/6 (days 2, 4, and 6, respectively) of the experimental phase. A milk sample of approximately 100 mL was collected, preserved with Bronopol (Schmehl Laborausstattung, Rostock, Germany), and stored at 4 °C until analysis. Milk samples were sent to the Milchkontroll- and Rinderzuchtverband eG (Güstrow, Germany) for the analysis of milk fat, milk protein, lactose, and milk urea by mid-infrared spectroscopy (MilkoScan, Foss GmbH, Rellingen, Germany) and somatic cell content (SCC) by flow cytometry (Fossomatic, Foss GmbH, Rellingen, Germany). The energy-corrected milk (ECM) yield was calculated from milk composition according to ECM (kg/d) = [0.038 × fat (g) + 0.024 × protein (g) + 0.017 × lactose (g)] × milk (kg/d)/3.14.

### Ruminal SCFA Composition

Rumen fluids were collected 4 h after the morning feeding on days −1, 3, and 6 by using an esophageal tubing system. The rumen fluid was subjected to pH measurement (CG 841, Schott, Mainz, Germany), passed through a 0.7 mm sieve, and centrifuged at 13,000*g* for 10 min at 4 °C. Subsequently, 2.5 mL of the supernatant was mixed with 1 mL of 0.5% iso-caproic acid (internal standard), and stored at −20 °C until further analysis. Rumen fluid samples were thawed, and 1 mL of sample was acidified with 5 μL of 37% hydrochloric acid. The concentrations of SCFA were analyzed in triplicate by a gas chromatograph coupled with a flame ionization detector (GC-FID, Series 17A; Shimadzu Corp., Kyoto, Japan) and equipped with a 25 m × 0.25 mm free fatty acid phase column (Roth, Karlsruhe, Germany).

### Proteome Analysis

In total, MG tissue samples from 6 cows per group were randomly assigned for proteome analysis. For protein extraction, 50 mg of tissue powder and 200 μL of lysis buffer consisting of Tris–HCl (50 mM; Carl Roth), EDTA (1 mM; GE Healthcare), NaF (10 mM: Thermo Fisher Scientific), IGEPAL CA-630 (1% v/v; Sigma-Aldrich), Triton X-100 (1% v/v), sodium deoxycholate (DOC; 0.5% v/v), sodium dodecyl sulfate (SDS; 0.1% w/v), and Roche complete Protease Inhibitor Cocktail tablet (one tablet per 10 mL of buffer; Roche Diagnostic, Mannheim, Germany) were homogenized for 45 s. Samples were centrifuged for 10 min at 4 °C and 16,100*g*. The protein concentration was measured using the Bradford method and bovine serum albumin as the standard. Protein extracts (25 μg per lane) were run on 15% SDS-PAGE. The resultant gel was stained with Coomassie brilliant blue (Serva Electrophoresis GmbH, Heidelberg, Germany) overnight and washed with distilled water. One lane per animal was cut into 10 slices (180 slices in total; Figure S2) and subjected to HPLC and mass spectrometry analysis. Each slice was transferred into a 1.5 mL reaction tube and washed twice with 100 μL of a solution with 50% CH_3_OH and 50% 50 mM NH_4_HCO_3_ for 30 min and once with 100 μL of 75% CH_3_CN for 10 min. Samples were dried at 37 °C for 20 min and incubated with 4 μg/mL trypsin solution overnight at 37 °C. For extraction, gel slices were covered with 60 μL of 0.1% trifluoroacetic acid in 50% CH_3_CN and incubated under shaking for 30 min. The peptide-containing supernatant was transferred into a clear glass vial and dried at 45 °C for 100 min in a concentrator (Eppendorf, Hamburg, Germany). The dry peptides (nonreduced or alkylated) were resuspended in 10 μL of CH_3_CN/H_2_O/trifluoroacetic acid (50%/49.5%/0.5%). Peptides were separated and analyzed using a Proxeon easy nLCII-system (Thermo Scientific) coupled to a Thermo Scientific LTQ Orbitrap-XL mass spectrometer. A 0.1 × 200 mm column with C18 Aeris Peptide (Phenomenex, Torrance, CA, USA) and a gradient of 0.5%/min (buffer A = 0.1% formic acid in water, Optima LC/MS; buffer B = 0.1% formic acids in 99.9% CH_3_CN, Optima LC/MS; Fisher Scientific) at a flow rate of 0.3 mL/min was applied. For MS and MS/MS analysis, a full survey scan in the Orbitrap-XL with a mass range (*m*/*z* 300–2000) and a Fourier transform resolution of 30,000 was followed by data-dependent fragmentation experiments of the most intense ions. Data were acquired in a data-dependent “top 5” format, selecting the most abundant precursor ions from the FTMS scan (mass range 300–2000 Da). The FTM scans were acquired with a resolution of 30,000 and a target value of 1.2 × 106 in the Orbitrap analyzer. The ion-trap MS scans were acquired with uni-mass resolution in the LTQ using 3000 as the target value, 2 as the default charge state, and a lower intensity threshold for MS2 of 3000 counts. The normalized collision energy in the collision-induced dissociation was 35 eV, and dynamic exclusion was defined by a list size of 500 with an exclusion duration of 30 s. The spectra were acquired in the LTQ via collision-induced dissociation. The parameters for the dynamic exclusion list are as follows: repeat count = 1, repeat duratio*n* = 30 s, exclusion list size = 500, and exclusion duration = 30. The mass spectrometry was deposited to the ProteomeXchange Consortium via the PRIDE^24^ partner repository. Data files were searched against the National Center for Biotechnology Information Bovine database (http://www.ncbi.nlm.nih.gov/) using Mascot version 2.6.2 with the common contaminant “Keratine” specified. The Mascot search was carried out considering the following parameters: parent ion mass tolerance of 10 ppm, fragment ion mass tolerance of 0.80 Da, and Met oxidation (+15.99492 Da).

Each Mascot search included the data from all 10 gel slices per lane and results loaded into Scaffold software (version 5.0.1., Proteome Software Inc., Portland, OR, USA). The Scaffold viewer was utilized to validate MS/MS-based peptide and protein identifications. Peptide identifications were accepted with two peptides characterizing uniquely one protein with a 95% probability of achieving a false discovery rate of less than 0.1% by the Peptide Prophet algorithm with Scaffold delta-mass correction (Table S1). Only proteins identified in at least 4 of 6 animals per group were considered for further analysis. For the analysis of differential protein expression, raw spectral counts were processed utilizing the DESeq2 package of the bioconductor repository in R (www.bioconductor.org). The raw spectral counts and the metadata were used to generate a DESeqDataSet object with DESeqDataSetFromMatrix (Table S1). The DESeq function performed estimate size factors, estimate dispersions, and negative binomial WALD test analysis with *p*-value criteria of 0.05. Differentially regulated proteins were subjected to functional enrichment analysis using Database for Annotation, Visualization and Integrated Discovery (DAVID, version 6.8 with updates from March 2023).^25^ The unique list of differentially expressed proteins with official gene symbols was submitted as a gene list and the *Bos taurus* database as the background. The cutoff value of the Benjamin–Hochberg factor was 0.05, and only the results from gene ontology (GO) analysis and Kyoto Encyclopedia of Genes and Genomes (KEGG) pathway analysis were selected for functional annotation categories (https://david.ncifcrf.gov/tools.jsp). KEGG pathways related to human diseases were excluded.

### RNA Extraction and RT-qPCR

From the same cows utilized for the proteomics analysis, RNA was extracted from 20 mg of MG tissue powder utilizing the innuPREP RNA mini kit and innuPREP DNase I (Analytik Jena, Jena, Germany). The RNA concentrations were measured by a NanoPhotometer (Implen, Munich, Germany). The RNA quality was determined with an Agilent 2100 Bioanalyzer (Agilent Technologies, Santa Clara, CA, USA). The RIN factors were between 6.6 and 7.9 (mean: 7.3). First-strand cDNA synthesis (1000 ng of RNA) was completed using a SensiFAST cDNA synthesis kit (Bioline, London, UK). Real-time qPCR was performed on a LightCycler 2.0 apparatus (Roche, Basel, Switzerland). Primers were designed using Primer3 software^26^ (v0.4.0). One PCR contained 2 μL of diluted cDNA (10 ng/μL), 1 μL of PCR-grade H_2_O, 0.5 μL of each primer (4 μM), and 6 μL of 2× Puffer SensiFAST SYBR No-ROX mix (Bioline) and was carried out in duplicate. The efficiency of amplification was calculated using LinRegPCR software^27^ (v2014.4; Academic Medical Centre, Amsterdam, The Netherlands), yielding efficiency values between 1.75 and 1.89 (Table S3). Data were quantified by qbase software (Biogazelle, Gent, Belgium) using peptidylprolyl isomerase A and eukaryotic translation initiation factor-3 subunit K (EIF3K) as reference genes (*M* value: 0.358; *V*-value: 0.125).

### Statistical Analysis

Periodically repeated measurements on the same animal were analyzed by repeated measurement ANOVA using the MIXED procedure of SAS (ver. 9.4, SAS Institute Inc., Cary, NC, USA). For milk composition and ruminal SCFA, the model contained the fixed-effects group (HS, CON, and PF), time (day of experimental phase), block (1 to 10), and the interaction between group × time, and DIM served as the covariate. For surface temperatures, the fixed effects were group (HS and CON), time (day 1 and day 6), block (2 to 10), and the group × time interaction, and milk yield served as the covariate. Repeated measures on the same animal were considered by the repeated statement of proc MIXED (repeated variable: time) using an autoregressive type or compound symmetry (based on lowest AIC) for the block diagonal residual covariance matrix. Least-square means (LSmean) and their standard errors were computed for each fixed effect in the ANOVA model. Additionally, differences of these LSmean values were tested using the Tukey–Kramer procedure. The SLICE statement of proc MIXED was used to perform a partitioned analysis of the LSmean for the interaction group × time.

## Results

### Body Surface Temperature and MG Characteristics

**Figure 2:**
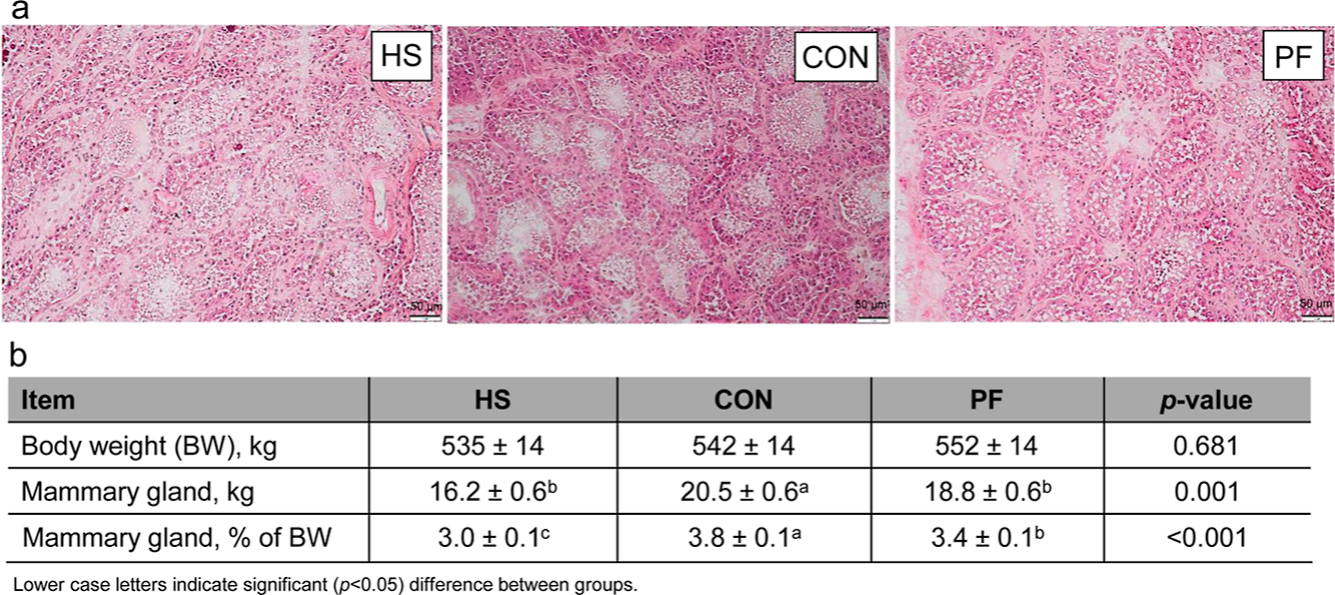
(a) Representative HE-stained MG tissue of heat-stressed (HS), control (CON), or pair-fed (PF) dairy cows. Scale bar 100 μm. (b) Body weight (BW) and udder weight after 7 days of challenge (*n* = 10 cows per group; LSM ± SEM).

Table 1 shows the mean, minimum (min), and maximum (max) surface temperatures of the lateral and posterior MG and the left abdominal site at which the rumen is located. The surface temperatures (including mean, min, and max) were significantly higher in cows kept for 6 days under HS conditions than at thermoneutrality (*p* < 0.05; Figure 1). Both the lateral and posterior MG had a greater maximum temperature than the lateral abdominal surface temperature during HS (lateral MG: 39.66 °C, posterior MG: 40.08 °C, and left abdominal surface: 37.67 °C). The difference between the left abdominal or the MG surface temperature and the ambient temperature was lower under HS in comparison to thermoneutral conditions (*p* < 0.05). After 7 days of challenge, the morphological analysis of HE-stained MG tissue, including morphological alterations of the MEC and luminal immune cells, revealed no difference between the groups (Figure 2). The MG weights of the HS and PF cows were lower than those of the CON cows (*p* < 0.01; Figure 2), while the MG weight as a percentage of body weight was lower in HS than in PF and CON cows (*p* < 0.001) but also lower in PF than in CON cows (*p* < 0.001).

**Table 1:** Body Surface Temperatures of Ad Libitum-Fed Dairy Cows Exposed to 28 °C (HS, *n* = 9) or 16 °C (CON, *n* = 9)^a^

		group					*p*-value		
		HS		CON					
item		day-1	day 6	day-1	day 6	SEM	group	time	group × time
lateral MG	mean °C	33.9	36.9	33.0	32.7	0.5	<0.001	<0.05	<0.01
	min °C	26.3	30.2	23.8	21.9	1.3	<0.001	0.498	<0.05
	max °C	38.1	40.1	38.0	38.1	0.4	<0.05	<0.05	<0.05
posterior MG	mean °C	35.3	36.9	34.6	33.9	0.5	<0.001	0.378	<0.05
	min °C	25.6	29.6	25.6	25.1	1.3	<0.05	0.279	0.137
	max °C	38.1	39.7	38.2	37.8	0.5	<0.05	0.297	0.081
rumen size confined left abdomen	mean °C	29.2	33.7	29.2	28.5	1.0	<0.05	0.110	<0.01
	min °C	31.5	31.5	31.7	31.6	0.6	<0.001	<0.001	<0.001
	ax °C	33.4	37.7	33.5	32.8	0.4	<0.001	<0.01	<0.001
mean body temperature	lateral udder, K	16.4	13.9	17.6	19.2	1.0	<0.001	0.704	0.052
minus ambient temperature	posterior udder, K	17.1	16.1	17.0	20.0	1.1	0.108	0.292	0.092
	rumen, K	15.4	9.0	15.8	15.9	0.5	<0.001	<0.001	<0.001

a Data are shown as LSM ± SEM.

### Milk Composition and Milk Yield

The day before the start of the experimental phase, milk yield and ECM yield were comparable between groups, but the percentage of milk fat was lower in HS than in PF cows (*p* < 0.05, Table 2). Milk yield, protein and lactose yield, energy (ECM), and fat (FCM)-corrected milk yield changed over time. From day 4 to 6 of the experimental phase, milk yield, and on day 6, ECM and FCM were lower in HS than in CON cows (*p* < 0.05). Furthermore, milk protein yield and lactose yield were lower in HS than in CON on days 4 and 6. On day 6, milk protein yield was lower in HS than in PF cows (*p* < 0.05), while protein and lactose percentages were unaltered among the groups. From day 3 to 6, the milk urea concentration was higher in HS than in CON and PF cows (*p* < 0.05), but the SCC did not differ among groups or over time. It must be noted that the differences and similarities in milk concentrations existed not only between the nine animals per group but also between the subset of animals (*n* = 6) used for proteome analysis (data not shown).

**Table 2:** Milk Yield and Composition of Heat-Stressed (HS), Control (CON), or Pair-Fed (PF) Dairy Cows (*n* = 10 Cows per Group)^a^

	group					*p*-value		
item	time	HS	CON	PF	SEM	group	time	group × time
daily milk yield, kg	–1	29.03	28.00	28.35	1.44	0.270	<0.001	<0.001
	2	26.25	28.55	28.48	1.44			
	4	22.54^b^	27.92^a^	25.39^a,b^	1.45			
	6	21.52^b^	27.38^a^	25.16^a,b^	1.45			
milk fat,%	–1	3.87^b^	3.66^a,b^	4.48^a^	0.023	0.076	<0.05	0.741
	2	4.01	3.70	4.14	0.023			
	4	4.30	3.94	4.61	0.023			
	6	4.12	4.02	4.58	0.023			
milk fat, kg/d	–1	1.12	1.02	1.25	0.07	0.115	0.108	0.051
	2	1.06	1.05	1.17	0.07			
	4	0.97	1.09	1.16	0.07			
	6	0.89^b^	1.09^a,b^	1.14^a^	0.07			
milk protein, %	–1	3.48	3.44	3.56	0.08	0.319	<0.001	<0.01
	2	3.36	3.39	3.52	0.08			
	4	3.22	3.33	3.40	0.08			
	6	3.12	3.31	3.37	0.08			
milk protein, kg/d	–1	1.01	0.95	1.01	0.05	0.131	<0.001	<0.001
	2	0.89	0.96	1.00	0.05			
	4	0.73^b^	0.92^a^	0.86^a,b^	0.05			
	6	0.67^b^	0.90^a^	0.85^a^	0.05			
lactose, %	–1	4.90	4.85	4.88	0.04	0.573	0.098	0.880
	2	4.94	4.86	4.91	0.04			
	4	4.89	4.85	4.88	0.04			
	6	4.89	4.86	4.92	0.04			
lactose, kg/d	–1	1.42	1.36	1.39	0.07	0.347	<0.001	<0.001
	2	1.30	1.39	1.40	0.07			
	4	1.10^b^	1.36^a^	1.24^a,b^	0.07			
	6	1.05^b^	1.33^a^	1.23^a,b^	0.07			
milk urea, mg/L	–1	223	222	235	21.2	<0.01	<0.001	<0.001
	2	315	264	252	21.1			
	4	403^a^	269^b^	237^b^	21.2			
	6	369^a^	248^b^	235^b^	21.2			
SCC, ×10^3^ cells/mL	–1	160	35	122	134	0.489	0.483	0.315
	2	207	25	104	133			
	4	38	23	109	134			
	6	245	106	88	134			
ECM, kg	–1	29.29	27.32	30.73	1.44	0.174	<0.001	<0.001
	2	26.98	29.94	29.74	1.43			
	4	23.60	27.86	27.70	1.44			
	6	21.87^b^	27.56^a^	27.33^a^	1.54			
3.5% FCM^b^, kg/d	–1	30.66	28.64	32.56	1.57	0.185	<0.001	<0.05
	2	29.47	28.50	31.28	1.61			
	4	25.49	29.67	29.79	1.58			
	6	23.7^b^	29.44^a^	29.40^a^	1.58			

a Data are shown as LSM ± SEM. Lowercase letters indicate significant differences between groups (p < 0.05). ECM—energy-corrected milk, FCM—fat-corrected milk, SCC— somatic cell count.

b 3.5% FCM = (0.4324 × kg of milk yield) + (16.216 × kg of milk fat yield).

### Ruminal pH and SCFA

Next, we analyzed ruminal SCFA concentrations because acetate is the main precursor for the de novo milk fat synthesis. Except for isovaleric acid, the molar portion of individual SCFA as well as the total SCFA concentrations and ruminal pH were not different before the start of the challenge (Table 3). During HS, the molar acetic acid portion increased over time (p < 0.05), whereas the portions for *n*-valeric acid (p < 0.01) and *n*-caproic acid (p < 0.05) decreased. As a result, the portion of acetate and the acetate/propionate ratio were greater in HS than in CON cows on days 3 and 6 (p < 0.05), and the acetate/propionate ratio was higher in HS than in PF cows on day 3 (p < 0.05). Furthermore, the molar portion of n-valerate was lower in HS and PF than in CON cows (p < 0.05, respectively), and *n*-caproic acid tended to be lower in HS than in PF cows (p = 0.09) on day 6. Ruminal butyrate was not different between the groups.

**Table 3:** Ruminal pH and Short-Chain Fatty Acid (SCFA) Composition of Heat-Stressed (HS), Control (CON), and Pair-Fed (PF) Dairy Cows (*n* = 10 Cows per Group; LSM ± SEM)^a^

	group					*p*-value		
item	time	HS	CON	PF	SEM	group	time	group × time
ruminal pH	–1	6.67	6.69	6.73	0.14	0.652	0.943	0.659
	3	6.69	6.58	6.77	0.13			
	6	6.78	6.56	6.77	0.13			
total SCFA [mM]	–1	85.96	89.41	74.16	8.13	0.479	0.306	0.947
	3	87.27	89.14	79.37	7.95			
	6	74.41	81.32	74.75	8.18			
acetic acid [mol %]	–1	58.84	58.41	58.81	1.02	<0.05	<0.05	<0.05
	3	62.02^a^	58.49^b^	59.75^a,b^	0.99			
	6	60.52^a^	55.99^b^	59.75^a^	1.02			
propionic acid [mol %]	–1	20.77	21.86	19.98	0.66	<0.05	0.363	<0.01
	3	18.50^b^	20.67^a^	21.50^a^	0.64			
	6	20.12^b^	22.25^a^	19.70^b^	0.66			
ratio	–1	2.87	2.72	2.98	0.13	<0.01	0.194	<0.01
acetic acid	3	3.39^a^	2.83^b^	2.80^b^	0.12			
propionic acid	6	3.08^a^	2.53^b^	3.06^a^	0.13			
iso-butyric acid [mol %]	–1	1.86	1.73	2.05	0.13	0.655	0.307	0.531
	3	1.74	1.82	1.83	0.13			
	6	1.92	1.89	1.94	0.13			
*n*-butyric acid [mol %]	–1	13.71	13.41	13.81	0.63	0.446	0.336	0.073
	3	13.42	14.15	12.39	0.61			
	6	13.07	14.76	13.86	0.63			
iso-valeric acid [mol %]	–1	1.71^b^	1.58^b^	2.06^a^	0.11	0.064	0.199	<0.05
	3	1.69^B^	1.92^A,B^	2.02^A^	0.10			
	6	1.64	1.87	1.71	0.11			
*n*-valeric acid [mol %]	–1	2.08	2.02	2.11	0.11	0.172	<0.01	0.083
	3	1.74	1.95	1.71	0.11			
	6	1.82^b^	2.20^a^	1.81^b^	0.11			
*n*-caproic acid [mol %]	–1	1.04	1.00	1.19	0.11	0.425	<0.05	0.175
	3	0.90	0.99	0.83	0.10			
	6	0.92^B^	1.04^A,B^	1.23^A^	0.11			

a Lowercase letters indicate significant (p < 0.05) and capital letters tending (0.05 > p < 0.09) differences between groups.

### Functional Classification of Differentially Expressed Proteins

Proteomic analysis identified between 1147 and 1457 proteins per group (Table S1). According to the sorting criteria, 880 proteins were abundant in all three groups. The comparison between HS and CON cows revealed 133 differentially expressed proteins (Figure S2), of which 59 were up-regulated and 74 were down-regulated (Table S1). Moreover, we found 33 differentially expressed proteins between HS versus PF cows (Figure S1), of which 20 were up-regulated and 13 down-regulated (Table S1). Comparing the PF vs CON group, 97 differently expressed proteins were found (Figure S1), of which 32 were up-regulated and 65 were down-regulated (Table S1). In addition, a total of 43 proteins were found in common between HS vs CON and PF vs CON cows, while the comparisons between HS vs CON and HS vs PF cows revealed 6 commonly shared proteins. Among them, apolipoprotein A4 (APOA4), aspartyl-tRNA synthetase (DARS), and prothymosin alpha (PTMA) were down-regulated, and 4-hydroxy-tetrahydrodipicolinate reductase (DapB), Parkinsonism-associated deglycase (PARK7), and ribosomal protein S27-like (RPS27L) were up-regulated in both HS vs CON and HS vs PF comparisons.

**Figure 3:**
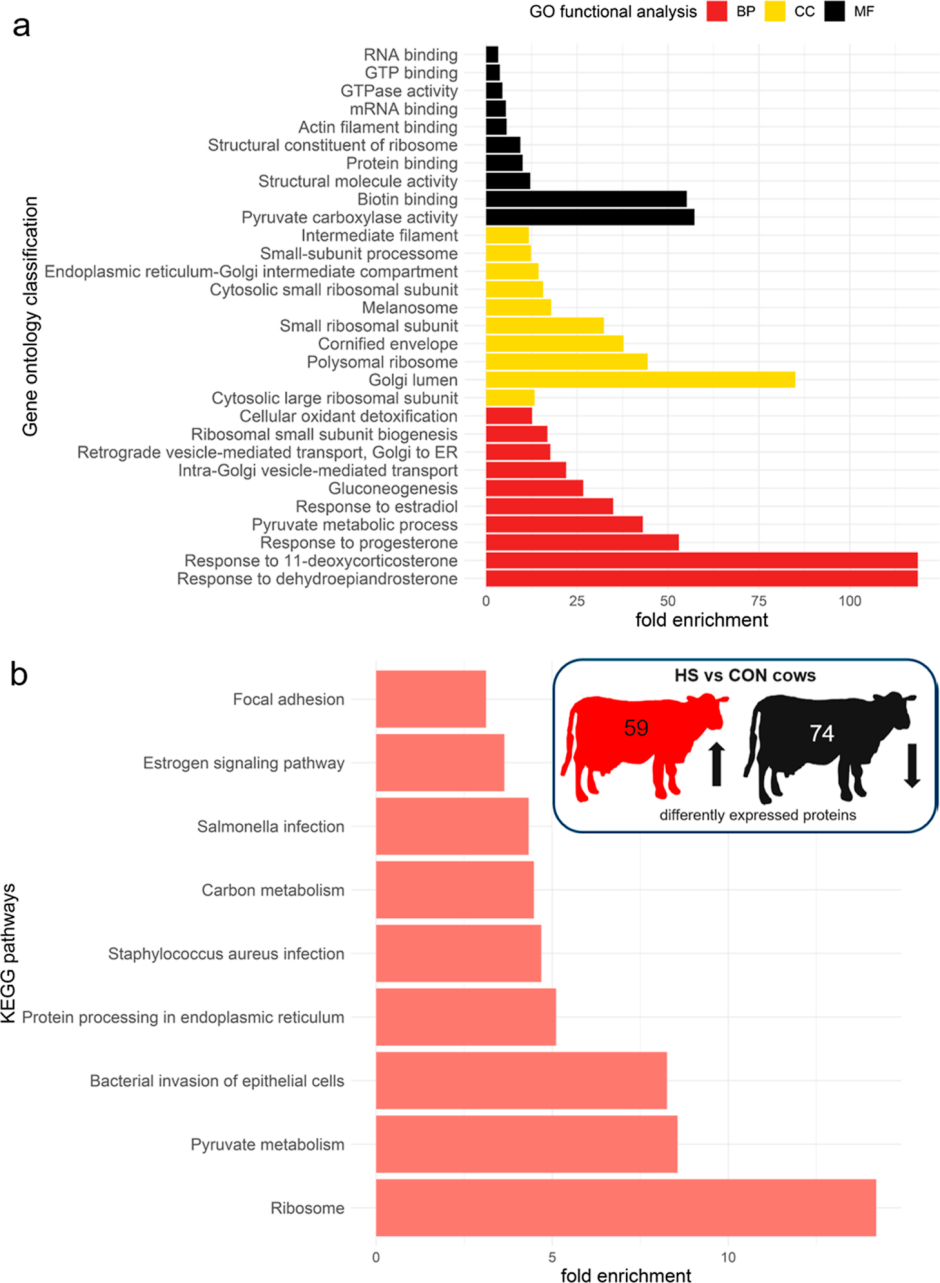
(a) Top 10—GO classification of proteins based on their involvements in BP, CC, and MF and (b) KEGG pathway enrichment analysis for HS vs CON cows (*n* =6 cows per group).

**Figure 4:**
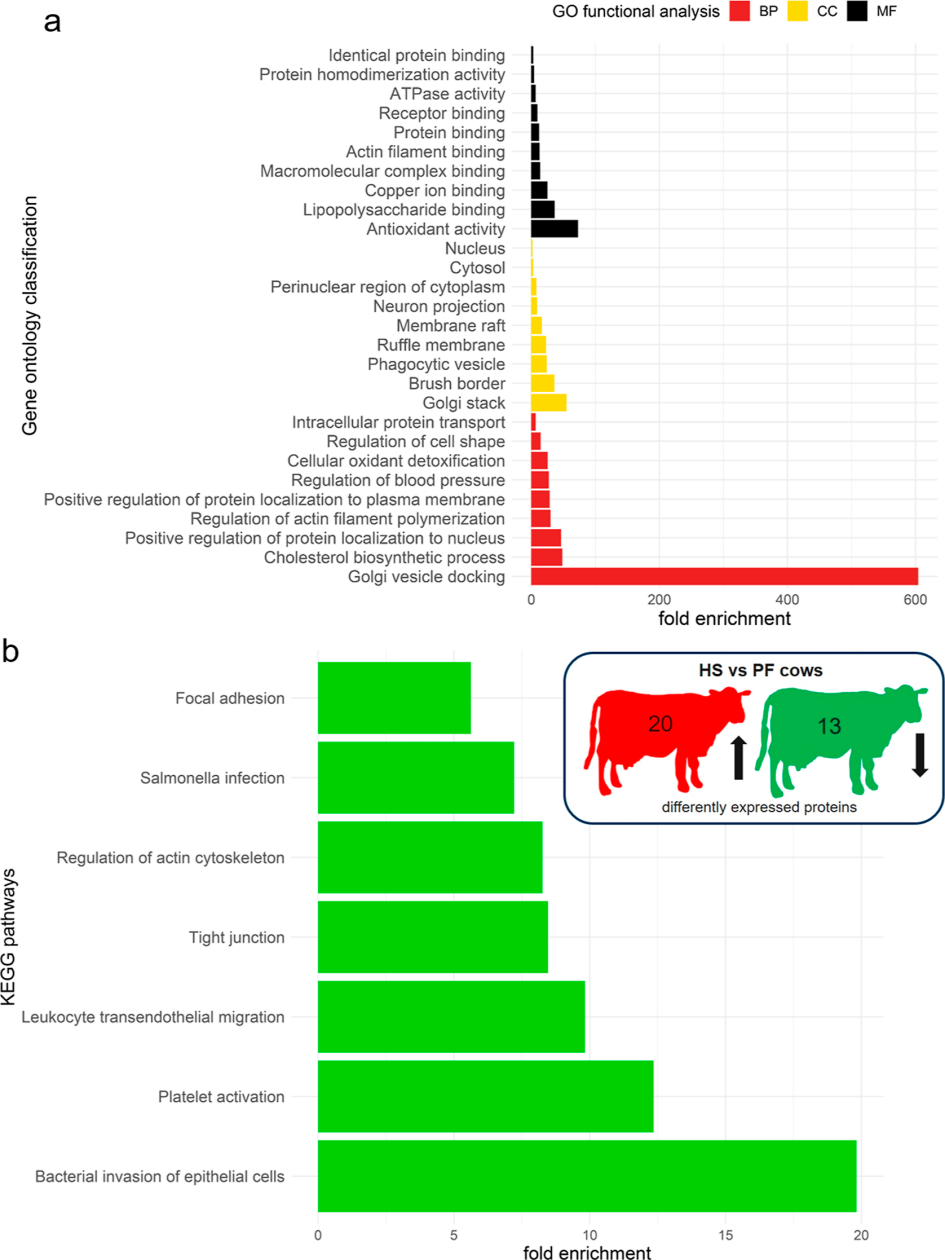
(a) Top 10—GO classification of proteins based on their involvements in BP, CC, and MF and (b) KEGG pathway enrichment analysis for HS vs PF cows (*n* = 6 cows per group).

To further characterize the molecular and cellular changes during HS, we performed GO and KEGG pathway analyses (Figures 3 and 4). The top 10 GO terms for the comparison between HS and CON cows show that cellular component (CC) changes occurred predominantly in the Golgi lumen, polysomal ribosome, and small ribosomal subunit (Figure 3A). The biological processes (BP) were mainly related to hormonal responses including dehydroepiandrosterone, 11-deoxycorticosterone, progesterone, and estradiol. In terms of the molecular function (MF), proteins involved in pyruvate carboxylase (PC) activity, biotin binding, and structural molecule activity were highly enriched in HS than in CON cows. The KEGG pathway analysis specified these results, highlighting the main differences between HS and CON cows in the ribosome and protein processing, carbon and protein metabolism, *Staphylococcus aureus* infection, and estrogen signaling (Figure 3B).

The comparison of HS and PF cows using GO revealed that the Golgi stack, brush border, and phagocytic vesicles were the dominantly affected cell components, and this finding is reflected by a strong enrichment of the BP involving Golgi vesicle docking, cholesterol biosynthesis, and regulation of protein localization to the nucleus (Figure 4A). Furthermore, proteins with a MF in antioxidant activity, lipopolysaccharide binding, and copper ion binding were also enriched in HS relative to PF cows. Pathways related to bacterial invasion, platelet activation, leukocyte transendothelial migration, tight junction, and regulation of the cytoskeleton were the most affected when comparing HS and PF cows, as recognized by KEGG analysis (Figure 4B). Pathways, commonly enriched in HS versus CON and HS versus PF comparisons, are bacterial invasion of epithelial cells, *Salmonella* infection, and focal adhesion (Figures 3B and 4B). Manual evaluation of the list of differentially expressed proteins (Table S1) further revealed that fatty acid synthase (FASN), involved in milk fat synthesis, and the casein synthases CSN1S1, CSN1S2, CSN2, and CSN3, all involved in milk protein synthesis, were found to be down-regulated in HS vs CON. On the other hand, CSN2 and CSN3 were less abundant in HS vs PF cows. Furthermore, the abundances of the fatty acid binding proteins (FABP) 3 and 5 were not altered by the treatments. Among the peptide or amino acid transporter, the protein expression of the solute carrier family 3 member 2 (SLC3A2) was also not affected by the treatment, while the sodium-dependent phosphate transport protein 2B (NaPi2b; encoded by the solute carrier family 34 member 2; *SLC34A2*) was significantly down-regulated in HS than in CON cows.

In addition, proteins involved in the pyruvate and energy metabolism, namely, PC, phosphofructokinase (PFKL), acetyl-CoA acetyltransferase 1 (ACAT1), fumarate hydratase (FH), and phosphoserine aminotransferase 1 (PSAT1), were down-regulated in HS vs CON but not HS vs PF cows. However, the expression of the monocarboxylate transporter 1 MCT1 (encoded by the *SLC16A1* gene) which regulates the lactate, pyruvate, acetate, and ketone bodies shuttling remained unaffected by ambient heat. The comparison of the citrate and energy metabolism of PF and CON cows shows that pyruvate dehydrogenase E1 component subunit alpha (PDHA1) and 2-oxoglutarate dehydrogenase (OGDH) were down-regulated, while succinate-CoA ligase subunit beta (SUCLG2) was up-regulated (Tables S1 and S2). Regarding the bacterial invasion of the epithelial cell pathway, Rac family small GTPase 1 (RAC1), caveolin 1 (CAV1), and actin-related protein 2/3 complex subunit 3 (ARPC3) were up-regulated, while vinculin (VCL) was down-regulated in HS vs CON cows. The integrin subunit beta 1 (ITGB1) was only up-regulated in HS vs PF cows (Tables S1 and S2). In the focal adhesion pathway, filamin A and B (FLNA and FLNB) were found to be down-regulated in HS vs CON cows (Tables S1 and S2).

### mRNA Expression

**Figure 5:**
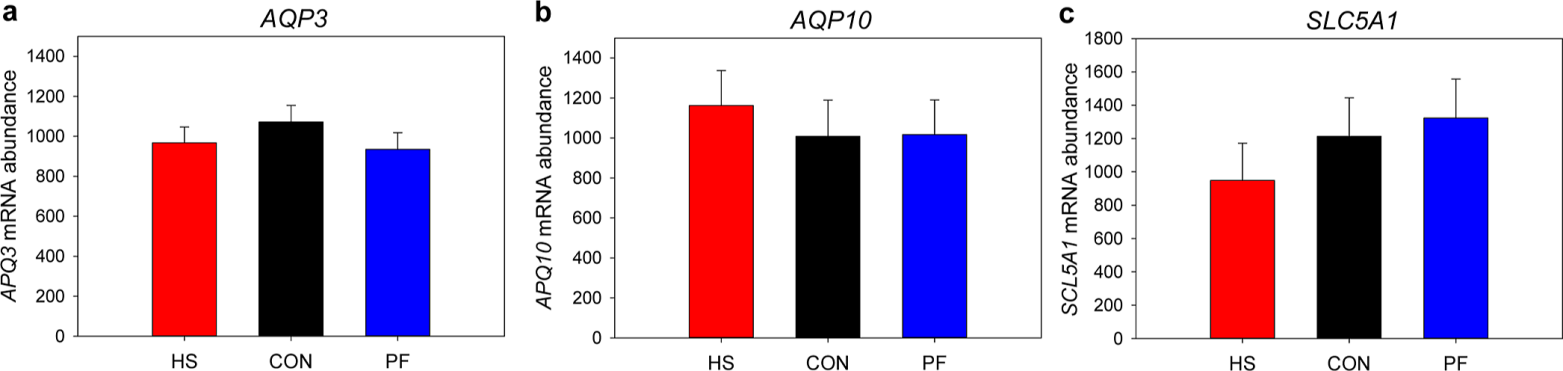
Relative mRNA abundance of (a) aquaporin 3 (*AQP3*), (b) aquaporin 10 (*AQP10*), and (c) solute carrier family 5 member 1 (*SLC5A1*) in MG tissue of heat-stressed cows (HS, red), cows receiving pair feeding (PF, blue), and control (CON, black) cows. HS cows were exposed to AT = 28 °C (THI = 76); PF and CON cows were kept at 16 °C (THI = 60) for 7 days (*n* = 6 cows per treatment; LSM ± SEM).

In order to assess the mRNA abundance of different transporter systems in the MG, aquaporin water channels (*AQP3*, *AQP10*) involved in the urea transport and the Na^+^-dependent glucose cotransporter (*SLC5A1*) were analyzed. Interestingly, none of the transporters differed between treatment groups (Figure 5).

## Discussion

HS has a tremendous effect on the performance of dairy cows. Integrating milk composition, ruminal SCFA, and functional analysis of the MG will help to better understand physiologically complex HS effects to apply sophisticated management to dairy cows. In the present study, we performed a HS trial in climate chambers to control the ambient temperature and humidity. We have previously described that HS cows of the present study had a higher rectal temperature, higher respiration rate, and lower dry matter intake during 7 days of HS in comparison to CON cows.^4^ To further assess the HS load in these cows, infrared thermography was performed before and 6 days after the start of thermal load to compare the surface temperatures of various body sites. Under both conditions, the surface of the MG showed the highest temperature. It is known that with the onset of lactation, vasodilation and blood flow increase among others to supply the MG with the required nutrients for milk production (estimation: 500 L blood for 1 kg of milk).^28^ With increasing ambient temperatures, the surface temperature of the lateral and posterior MG rose, likely due to an increased blood flow toward the periphery to dissipate endogenous heat, which can be as high as 388 W/m.^2,28^ The thin skin, low hair density, absence of subcutaneous fat, and shielded heat dissipation by the hind legs explain the higher surface temperature of the MG in comparison to other body compartments, e.g., the left abdominal site confining the left rumen site, although the rumen generates a high amount of fermentative heat.^29^

It is well known that the milk yield of lactating cows declines during HS. The milk yield decreased by 0.2 kg per unit THI increase when the THI was ≥72.^30^ The current study showed also a decrease in milk yield in HS compared with CON cows from 4 days of challenge on, which amounted to a 1.3 kg per unit THI increase above a THI of 72. Although not statistically different, the milk yield of HS was 2.8 kg lower after 4 days and 3.6 kg lower after 6 days of challenge than in PF cows. However, the ECM and FCM were significantly lower in HS than in CON and PF cows after 6 days of challenge, indicating a strong impact of HS on the concentration of milk constituents. Interestingly, variations in milk protein and fat concentrations depend on the duration of HS, THI, parity, milk yield, and geographical location of the dairy facility.^14^ In the present study, milk fat, protein, and lactose percentages were not different between groups on individual days of challenge, but we found a significant group × time effect for milk protein concentration. Our data are supported by previous findings, showing that the αs_1_-casein percentage in HS cows was lower than that in CON cows during the 9 day challenge period, while β- and κ-casein percentages were not affected by climate conditions (range from THI 72 to 78).^31^ However, a detailed analysis of casein isoforms could not be performed in the present study. The lower milk protein and fat yields of HS compared to CON cows on day 6 after challenge are likely due to lower protein expression of milk casein synthases (CSN1S1, CSN1S2, CSN2, and CSN3) and fatty acid synthase (FASN), respectively.

The milk urea concentration was higher in HS than in both control groups, indicating increased skeletal muscle protein degradation and proteolysis, with the latter stimulating hepatic amino acid deamination and urea synthesis.^6,8,32,33^ The urea transport from plasma into milk is mainly controlled by facilitated diffusion.^34^ However, we could not identify any known urea transporter by our proteomic approach; therefore, additional gene expression analysis was performed. The mRNA abundance of aquaporin water channels, namely, *AQP3* and 10, which are known to be permeable to water, glycerol, and urea,^35^ was found not affected by HS. A previous study examining low and high milk urea-excreting cows concluded that urea concentrations in milk are not directly controlled by urea transporters but rather by passive diffusion from the blood.^34^ Furthermore, a strong correlation between plasma urea and milk urea concentrations was found.^34,36^ This is in agreement with our study where HS cows have in parallel higher plasma and milk urea concentrations.^37^

The synthesis of milk constituents is predominantly dependent on the delivery of precursors, mainly SCFA, glucose, and amino acids. HS, however, limits the availability of these nutrients due to a decrease in feed intake and the prioritized allocation to vital functions.^38^ It has been shown that the total concentration of ruminal SCFA of nonlactating cows declines at 37 °C ambient heat and that this effect is not dependent on the level of feed intake because it is controlled through the cannula the animals are equipped with.^11,39^ Here we report that the total ruminal SCFA concentration of our cows was not affected by exposure to 28 °C or reduction in feed intake for up to 6 days. These results are in line with those reported by Bedford et al., who showed that total SCFA production is comparable in Holstein heifers exposed to 30 °C or when they are pair-fed for 10 days.^11^ However, we found a higher molar percentage of acetate after 3 days of challenge in HS compared with CON cows and after 6 days of challenge in HS and PF than in CON cows. Although PF animals consumed most of their diet immediately after the morning and afternoon feeding, HS animals ingested small portions throughout the day. This different feeding behavior may have an additional influence on the VFA composition, despite the same sampling period. On the other hand, the molar percentage of propionate was lower and the acetate/propionate ratio was greater in HS and PF than in CON cows, suggesting a shift in the microbial activity and fermentation of structural carbohydrates in response to reduced feed intake.^32^ Similarly, the propionate production and the acetate and propionate absorption changed in the same range no matter if heifers were transferred from thermoneutral conditions to either 30 °C or corresponding pair feeding.^11,39^ These and our results indicate that the changes in ruminal acetate and propionate concentrations are caused by the lower feed intake in HS and PF animals but not by increased ambient heat. In addition, the greater portion of acetate in HS and PF compared to CON cows cannot be explained by the direct or indirect interconversion of butyrate to acetate as suggested earlier^11^ because we did not find differences in ruminal butyrate concentrations between groups in the present study. Although not analyzed in the present study, it has been shown that the absorption of acetate from the rumen increases in HS and PF heifers relative to the thermoneutral situation,^11^ whereas the plasma acetate concentration decreases with reduction in feed intake.^33^ Based on these findings, we conclude that the greater portion of ruminal acetate cannot compensate for the loss in milk fat yield in HS compared to CON cows and that the lowered FASN expression of the MG is involved in this adaptive process. Interestingly, HS and PF cows had comparable portions of ruminal acetate on day 6 of the challenge, but PF cows showed a greater milk fat yield than HS cows. It has been shown that feed energy restriction initiates lipolysis, resulting in an increase of plasma long-chain fatty acid concentrations,^33^ which are extracted by the MG to be used for milk triglyceride synthesis.^9^ Therefore, the greater milk fat yield of PF than CON cows is likely achieved by the use of endogenous long-chain fatty acids overcompensating for the shortage in acetate supply for milk fat synthesis. However, SCFA other than acetate can also be used for the synthesis of milk fat, e.g., *n*-valeric acid and *n*-caproic acid, but although the molar portion of the latter is lower in the rumen of HS than in CON cows, it only contributes a minor portion to the total milk fat and has only low energetic importance.^40^ On the other side, the lower portion of ruminal propionate in HS compared to CON cows might be one reason for the lower milk lactose yield because propionate serves as a precursor for glucose production via hepatic gluconeogenesis and glucose is required for milk lactose synthesis. Besides, the immune system of HS cows has a great demand for glucose, further reducing the provision of glucose for milk lactose synthesis.^3^ Regarding the nutrient transport into the MEC, we found proteins involved in the transport of fatty acids (FABP 3 and 5), monocarboxylates (MCT1), amino acids/small peptides (SLC3A2), and the mRNA abundance of the glucose cotransporter *SLC5A1* not affected by HS. Similarly, the mRNA expression of the glucose transporter-I of the MG did not differ between noncooled cows and cows cooled for 7 and 56 days, further indicating that nutrient transporters are not strongly affected by thermal heat.^14^ The only transporter that was down-regulated on protein levels in HS than in CON cows was the pH-sensitive NaPi2b, suggesting that HS affects not all transport systems equally and that the milk phosphorus concentration could be altered in HS cows. However, more research is required to understand the complex nutrient shuttling into MEC during HS.

Our proteome analysis revealed that MG proteins related to pyruvate and energy metabolism (PC, FH, PSAT1, and ACAT1) were less expressed in HS than in CON cows. While PC catalyzes the carboxylation of pyruvate to oxaloacetate, FH facilitates the reaction of fumarate to l-malate, with the latter serving as a substrate for the synthesis of oxaloacetate.^41^ Previous studies reported that HS cows have decreased expression of malate dehydrogenase, an enzyme of the TCA cycle converting malate to oxaloacetate.^21^ Thus, our and previous^21^ findings suggest a diminished synthesis of oxaloacetate and NADH during HS. In addition, ACAT1, catalyzing the last step in β-oxidation of long-chain fatty acids, was expressed in HS lower than in CON but not in PF cows. Thus, it seems that β-oxidation in the MG of HS cows is reduced, whereas it functions in CON and PF cows. This conclusion agrees with the result of our earlier study demonstrating an increase in whole-body fat oxidation when ad libitum-fed cows at 15 °C were transferred to pair feeding, whereas this effect was blunted when cows were transferred to 28 °C.^42^ Further evidence of blunted fat oxidation was shown by Wheelock et al. (2010).^5^ Plasma nonesterified fatty acid (NEFA) concentrations were not altered during a 7 day HS trial, whereas NEFA concentrations increased in PF cows, indicating that lipolysis and fat mobilization were activated only by restricted feeding under thermal neutral conditions.^5^

In the citrate cycle and energy metabolism, PDHA1 and 2-oxoglutarate dehydrogenase (OGDH) were found to be down-regulated, while succinate-CoA ligase GDP-forming subunit beta (SUCLG2) was up-regulated in PF in comparison to CON cows kept at thermoneutral conditions. To conclude, the regulation of MG metabolism could partially explain the lower milk yield and altered milk composition during HS.

During the summer season, dairy cows are more prone to mastitis because the higher temperature and humidity create favorable conditions for bacterial infections of the MG, e.g., by streptococci and *Escherichia coli*, accompanied by an increase in SCC.^43^ In our 7 day trial, the SCC, an indicator for MG health, was not affected by the thermal load. However, pathways related to bacterial invasion of epithelial cells, leukocyte transendothelial migration, *S. aureus* infection, or *Salmonella* infection were enriched in HS cows compared to CON or PF cows, indicating an inflammatory response in the MG despite unchanged SCC during HS. Presumably, this inflammatory response provided an immune defense able to suppress signs of subclinical mastitis. Among the up-regulated proteins in HS cows were RAC1, CAV1, and ARPC3, all involved in G-protein-coupled signaling activating phagocytosis^44,45^ or Ras/Raf/Mitogen-activated protein kinase/extracellular-signal-regulated kinase (RAS-ERK) signaling of T-cell-mediated endocytosis,^46^ mediating the elimination of potential pathogens in MG tissue. In addition, ITGB1 was found to be up-regulated in HS compared to PF cows. Earlier studies demonstrated that integrins play an important role in the adhesion-induced phosphorylation and regulation of cell adhesion and cell trafficking of leukocytes in inflamed tissues,^47,48^ indicating activation of leukocyte migration during heat load, potentially to fight infection at its earliest stage. Interestingly, the immune function, e.g., of myeloid cells, depends on the correct expression of cell adhesion proteins.^49^ Among them, VCL plays an important role in actin cytoskeleton dynamics and integrin signaling. The down-regulation of vinculin might indicate an alteration in cell–surface regulation for cell adhesion affecting immune cell function.^49^ Further studies are required to uncover the complex regulatory function of immune cells during the heat load.

HS has been described to alter the morphology of the MG through inflammatory lesions, alveolar lumen appearance, fibrosis, or parenchymal destruction.^43^ However, morphological changes in the epithelium were not found during mild HS in our study. Regarding the KEGG pathway enrichment analysis, focal adhesion was found in both comparisons between HS and CON/PF cows. The down-regulation of FLNA and FLNB was found in HS compared with CON cows, indicating a thermal effect on cell–cell contact, cell shape, and migration.^50,51^

Of note, in the proteomic analysis, many proteins, e.g., ITGB1, thrombin (F2), COL1A1, and MY12B, were associated with platelet activation in HS cows compared with PF cows. As mentioned above, ITGB1 is involved in cell adhesion and also in the recognition of CD29 and fibronectin receptors,^52^ while the coagulation factor F2 plays an important role in thrombosis by converting fibrinogen to fibrin during blood clot formation.^53^ So far, platelet activation and the coagulation cascade have been described as conspicuous parameters during severe HS, inducing disseminated intravascular coagulation and leading to multiorgan dysfunction and death of the cow.^54^ Whether and how mild HS affects platelet activation and coagulation in the circulation requires further investigation.

To conclude, our data suggest that HS affects ruminal fermentation independent of the reduction of DMI by favoring ruminal acetate over propionate synthesis. However, we cannot exclude the possibility that reduced ruminal acetate absorption may explain the lower milk fat yield of HS cows. The altered availability of SCFA for the synthesis of milk in ambient heat is linked to the lower abundance of relevant proteins in the milk protein and fat synthesis process of the MG tissue, thus resulting in lower milk protein and fat yields. Furthermore, HS of the MG is accompanied by an adaptation of the pyruvate and carbon metabolism due to lower precursor availability with reduced feed intake, while bacterial invasion of epithelial cells, leukocyte transendothelial migration, and focal adhesion indicate a mild activation of inflammatory processes without signs of subclinical mastitis during mild HS. Our results highlight adaptive immune and metabolic responses to mitigate the negative effects of ambient heat in the MG. More research is required to validate platelet activation in the MG to understand blood clot formation in the whole body during HS.

## Data Availability

The mass spectrometry proteomics data have been deposited to the ProteomeXchange Consortium via the PRIDE partner repository^24^ with the data set identifier PXD048796.

## Supporting Information Available

Primer sequences; Venn diagram showing the number of differentially expressed MG proteins between heat-stressed (HS), control (CON), and pair-fed (PF) dairy cows (*n* = 6 per group); representative image of Coomassie-stained gel before the collection of the different bands for proteomic analysis; top 10 GO classification of proteins on the basis of their involvements in BP, CC, and MF; and KEGG pathway enrichment analysis between HS vs CON cows (*n* = 6 cows per group) (PDF)
Up- and down-regulated proteins between HS vs CON, HS vs PF, and PF vs CON dairy cows (XLSX)
KEGG pathways between HS vs CON, HS vs PF, and PF vs CON dairy cows (XLSX)
