# Supplementary material for: Novel Perspective on Molecular and Cellular Adaptations of the Mammary Gland-Regulating Milk Constituents and Immunity of Heat-Stressed Dairy Cows
Source: J Agric Food Chem. 2024 Sep 3;72(37):20286–98. doi: 10.1021/acs.jafc.4c03879 (PMC11421017; doi:10.1021/acs.jafc.4c03879)
Supplement: Supplementary file 1 — jf4c03879_si_001.pdf [file jf4c03879_si_001.pdf]

Supplemental Material to:

Novel perspective on molecular and cellular adaptations of the mammary gland regulating milk constitutes and immunity of heat stressed dairy cows

by Franziska Koch, Dirk Albrecht, Elke Albrecht, Christiane Hansen, Björn Kuhla

Supplementary Table 3. Primer sequences.

Gene		Primer sequence (5' to 3')	GenBank accession no.	Product size (bp)	Annealing (°C)	PCR efficiency	Ref.
AQP3	forward	CGCGAGCCCTGGATCA	NM_001079794.1	103	60	1.84	1
	reverse	CCCAGATCGCATCGTAATACAA					
AQP10	forward	TCCTGGCCGACATGCTATC	XM_024989821.1	101	60	1.89	2
	reverse	GCCCCAGCCAGCTACGTA					
SLC5A1	forward	TGTCCCTGGTGCTGTACATTT	NM_174606.2	130	60	1.89	2
	reverse	TAAAGGGCGGTGATTGCCA					
EIF3K	forward	CCAGGCCCACCAAGAAGAA	NM_001034489	125	60	1.75	3
	reverse	TTATACCTTCCAGGAGGTCCATGT					
PPIA	forward	GGATTTATGTGCCAGGGTGGTGA	XM_001252497	120	60	1.83	4
	reverse	CAAGATGCCAGGACCTGTATG					

Supplemental Figure 1. Venn diagram showing the number of differentially expressed mammary gland proteins between heat-stressed (HS), control (CON) and pair-fed (PF) dairy cows (n = 6 per group). Proteins, commonly differentially expressed between HS vs CON and HS vs PF, are named in the rectangle.

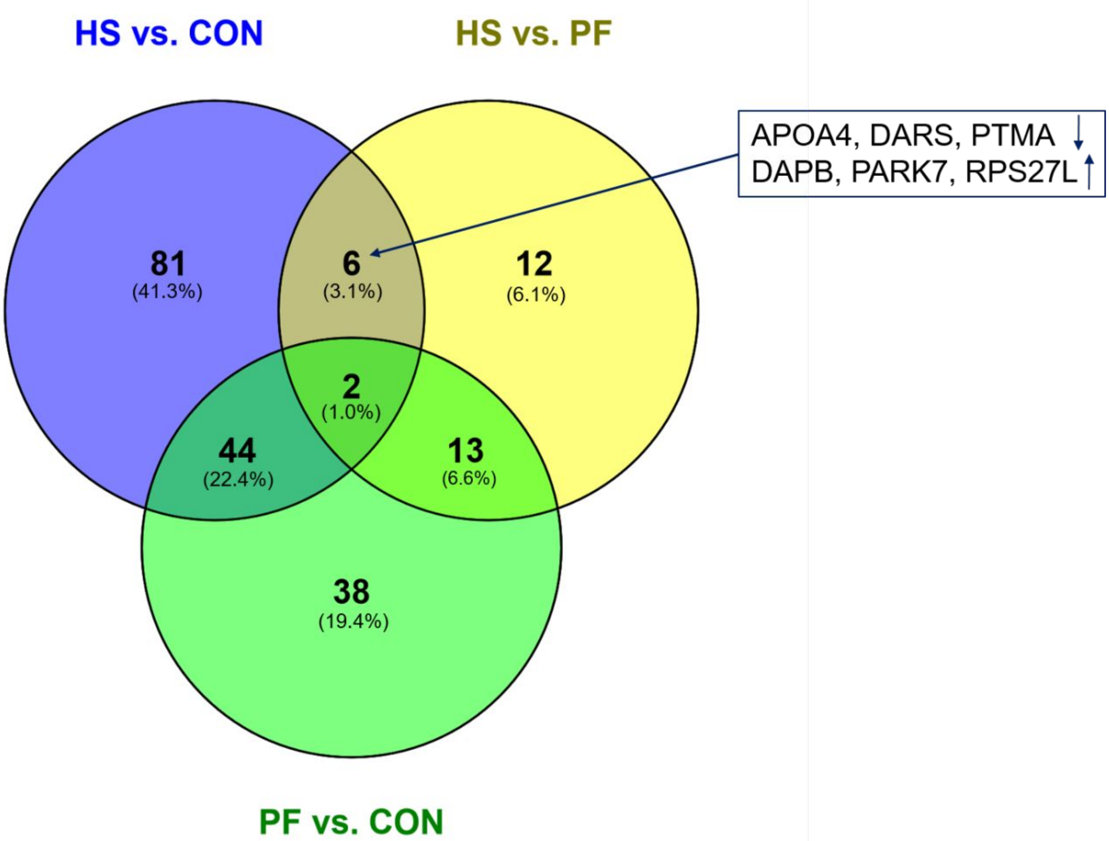

Supplemental Figure 2. Representative image of Coomassie stained gel before the collection of the different bands for proteomic analysis.

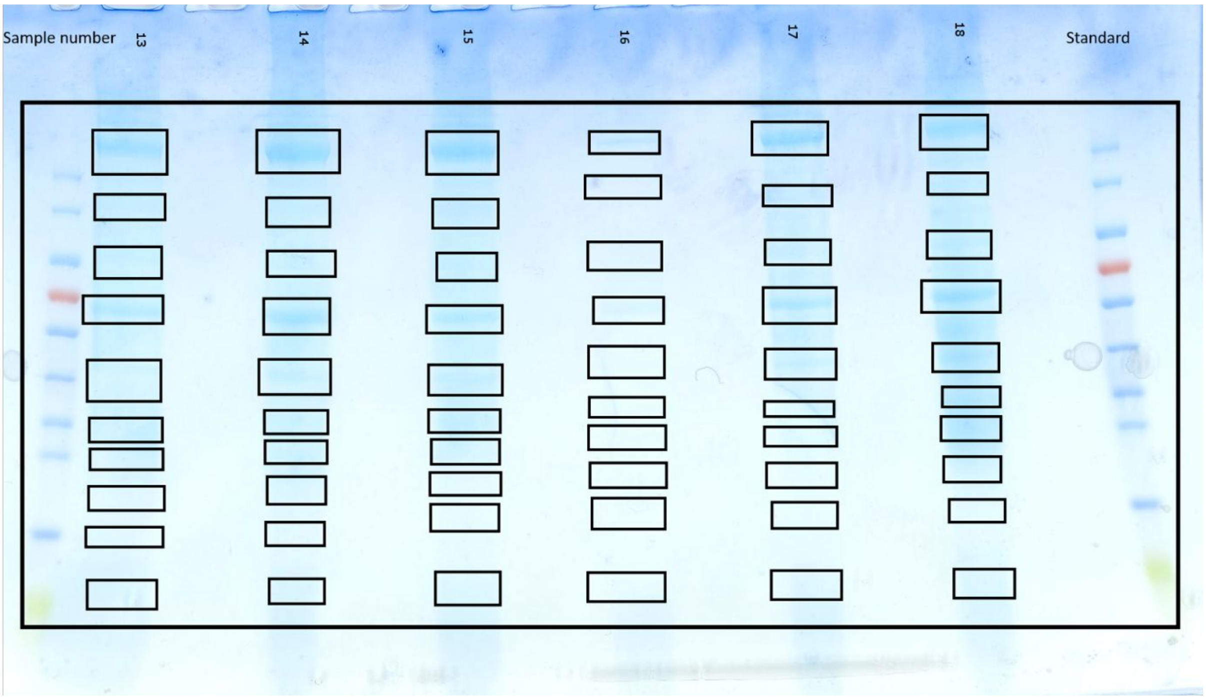

Supplemental Figure 3. (a) Top 10 - Gene ontology (GO) classification of proteins on the basis of their involvements in biological processes (BP), cellular component (CC) and molecular function (MF), and (b) KEGG pathway enrichment analysis between PF vs CON cows (n = 6 cows per group).

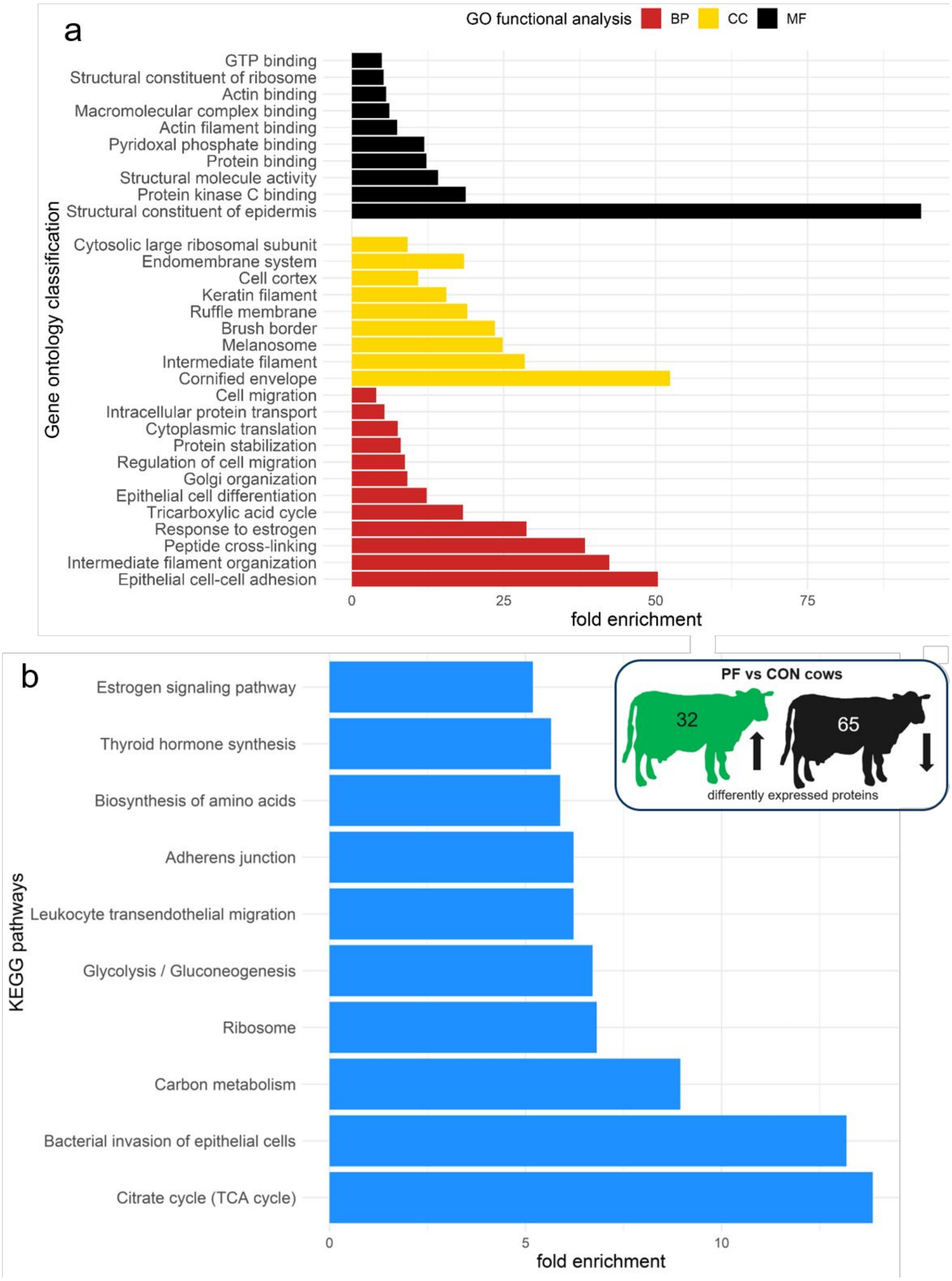

References

1. Rojen, B. A.; Poulsen, S. B.; Theil, P. K.; Fenton, R. A.; Kristensen, N. B., Short communication: Effects of dietary nitrogen concentration on messenger RNA expression and protein abundance of urea transporter-B and aquaporins in ruminal papillae from lactating Holstein cows. *J Dairy Sci* **2011,** *94* (5), 2587-91.
2. Koch, F.; Albrecht, D.; Gors, S.; Kuhla, B., Jejunal mucosa proteomics unravel metabolic adaptive processes to mild chronic heat stress in dairy cows. *Sci Rep* **2021,** *11* (1), 12484.
3. Kadegowda, A. K.; Bionaz, M.; Thering, B.; Piperova, L. S.; Erdman, R. A.; Loor, J. J., Identification of internal control genes for quantitative polymerase chain reaction in mammary tissue of lactating cows receiving lipid supplements. *J Dairy Sci* **2009,** *92* (5), 2007-19.
4. Bonnet, M.; Bernard, L.; Bes, S.; Leroux, C., Selection of reference genes for quantitative real-time PCR normalisation in adipose tissue, muscle, liver and mammary gland from ruminants. *Animal* **2013,** *7* (8), 1344-53.
